# Information needed for optimal immunization related to medical advice: an observational prospective cohort study protocol (INFORMed)

**DOI:** 10.3389/fpubh.2024.1481942

**Published:** 2024-11-22

**Authors:** Jennifer Wrenger, Bettina Berger, David D. Martin, Ekkehart Jenetzky

**Affiliations:** ^1^Institute for Integrative Medicine, Department of Medicine, Faculty of Health, Witten/Herdecke University, Witten, Germany; ^2^Department of Paediatrics, University Children’s Hospital Tübingen, Tübingen, Germany; ^3^Department of Pediatric and Adolescent Psychiatry and Psychotherapy, University Medical Centre, Johannes Gutenberg University Mainz, Mainz, Germany

**Keywords:** vaccination, infants, informed choice, shared decision-making, advice, health literacy

## Abstract

**Introduction:**

Today, accessing information on health issues is easier than ever. However, the flood of information can make decision-making difficult. Information can influence the intention for an action, yet the action often remains unpredictable. It is unclear if there is a relationship between the intention behavior gap and the wish for medical advice in parents of newborns as they have to deal with a number of vaccinations more than any other group of people. According to survey data, vaccine-hesitant people have less interest in vaccine advice.

**Methods and design:**

This study aimed to validate and elaborate this finding in a specific population and in a prospective observational manner. This study protocol was registered: https://drks.de/search/en/trial/DRKS00030716, DRKS00030716. The specific objectives include a primary endpoint focused on the wish for advice among hesitant and non-hesitant parents. Secondary endpoints involve comparing parents in terms of their respective information needs, which will be assessed based on: (a) vaccination attitudes at 6 weeks, (b) actual action taken at 12 weeks, and (c) the consistency of their attitudes and decisions. Parents of infants up to 6-week-old will be recruited and asked before the first recommended vaccination period and thereafter when the infant is 12 weeks old. Participants will receive an online questionnaire focusing on the information and advice they would like to receive and have received. Vaccination attitudes will be assessed using the C7C questionnaire at 6 weeks and the actual action of taking the first vaccine at 12 weeks.

**Discussion:**

INFORMed will provide data on information needs and wishes of young parents depending on their attitude toward vaccination. Based on the results, health literacy in parents can be improved and information strategies can be adapted.

## Introduction

It has never been easier to access information on any topic than it is today. Social media, in particular, offers many avenues for finding information, which can either undermine or reinforce one’s opinions. Evaluating the correctness of this information can be difficult, leading to uncertainty and confusion, especially in medical contexts. Patient preference is, next to expert clinical experience and current state of research, one of the three pillars of evidence-based medicine, but evidence for information strategies is very low and often focused on the view of healthcare experts. Many patients do wish to participate in the decision-making process (1, 2), yet information (3) and evidence show that especially hesitant patients tend to need individualized participatory formats (4). The decision process and way of education are based on different influencing factors and experiences.

Different strategies of participative decision-making have been developed over the years, which have in common that they can be time-consuming (5). Physicians are often working under time constraints, a factor that can contribute to limiting the extent to which they practice shared decision-making (6).

On the parents’ side, personal experiences influence their decision. As highly effective drugs, vaccines also have the potential to produce negative side effects that, although presumed to be rare (7), can be classified as threatening by parents (8). In this context, a lack of information seems to have a particular influence on decision-making to the effect that healthcare workers who provide more information are experienced, as being more trustworthy, resulting in a higher rate of vaccination (9). In some contexts, hesitant parents seem to wish less for information than non-hesitant parents (1). Good communication, which includes parents feeling understood (10), is then all the more important to answer open questions and to explain the current state of research (11). Even though there is research on shared decision-making and other forms of patient participation, it generally focuses on large population groups (1, 12) and seldom measures actual vaccine uptake (13, 14). Another difficulty with research in this area is that communication about vaccines cannot be compared between different vaccines as they cause different degrees of hesitancy (15).

The timing of the consultation could also play an important role, particularly in connection with childhood vaccinations. As immunization of infants is recommended as early as possible (16), one of the first decisions parents must take for their children after birth is whether they want to vaccinate as recommended or not, especially given that different countries have different recommendations. At no other time of life do humans face so many immunizations against as many pathogens as in the newborn phase and first year of life, on top of many other decisions, that this phase of family life requires. Hence, parents have to deal with much information at this time, and the vulnerable postpartum period can pose an extra strain on decision-making (16). Vaccinations are applied multiple times, but the most important is the first initial application, because parental experience of the first vaccinations may influence all following actions for their child (17). It is therefore particularly important to consider this phase of the first vaccination decision and make it as smooth as possible for the parents.

Considering these aspects, the study aims to ask new parents who they trust and what they need to make an informed and satisfying decision for their newborns. To improve research in vaccine promotion, parental attitudes must be investigated before and after the actual action of the initial vaccination consultation, which consists of two parts: the immunization advice and the initial vaccination itself. Hence, the immunization advice will be investigated in this study from the parental perspective. Furthermore, it is unclear what factors influence actions deviating from the parental vaccination attitude. Vaccine promotion has often the primary intention to persuade hesitant parents/patients and not the main aim to answer the expressed information needs of parents/patients. This implicit intention of each counseling session must be considered in analyses. This study is the first to distinguish these two intentions (persuasion and information satisfaction) in order to evaluate success in terms of the desire for counseling and actual vaccination. Therefore, parents will be asked before their first advisory and vaccination appointment about their needs and their attitude and after the first recommended time for vaccination about their experience, their action, and their suggestions for improvements. Since time management plays a major role in medical offices, one part of this study also deals with the search for information channels other than medical ones. The results may allow us to develop the underlying framework and promotion initiatives.

### Strengths of the study

- Exclusive focus on the population with the highest vaccination rate
- Prospective and longitudinal
- Differentiation between attitude and action
- Differentiating between vaccination education in principle and information content
- Information content checklist developed based on qualitative preliminary work (2)
- Neutrally observing and not intervening, thus also reaching hesitant parents (4)
- Theoretical framework examined in detail.

## Methods and analysis

This protocol is built in accordance with the STROBE guidelines (18) because it is a purely observational study.

### Study design

In contrast to most cross-sectional surveys with various ages, this investigation is planned (a) very early after the birth of the child and (b) as a prospective longitudinal study before and after the first vaccination. This purely observational study will exclusively focus on the parental view and does not entail a specific intervention. It is intended to assess requirements and needs for information and optimal vaccination intervention.

To minimize the stress associated with becoming a parent and expectation bias in the parent–pediatrician interaction, the study is designed as an online case report form, so that parents can easily answer the questions independently. This study is parent-based because the parents’ view is crucial for their decision.

### Sample selection

Physicians, midwives, social workers, and other professionals working with parents and parents-to-be register on the study website. For external validation, multiplicators answer a case report form that includes information necessary for the shipping of flyers, the profession of the multiplicator, and an adapted version of the 7C questionnaire to assess their attitude to vaccines.

Multiplicators hand out flyers to possible study participants and draw attention to the study. Depending on the professions of the multiplicators, flyers can be handed out to families with newborns younger than 6 weeks, for example, at the appointment for the U3 (a routine preventive medical checkup in Germany at the age of 4 to 6 weeks) or to expectant parents, for example, during preventive pregnancy checkups. Further advertising can take the form of posters, newsletters, or websites of multiplicators. If this way of recruitment does not result in sufficient participants, the use of less validated methods such as the distribution of the study website via social media could be discussed.

### Inclusion and exclusion criteria

As expectant and new parents get informed electronically via the study website, register and consent electronically in a separate REDCap database (19, 20), and participate via email, study participation is only possible for parents who have access to a digital device and have an email address. To guarantee consistent conditions, all participants need an affiliation with the German healthcare system. Furthermore, sufficient knowledge of the German language is needed.

Infants that are older than 8 weeks or families with one child already participating in the study will be excluded.

### Data collection

The registration process and consent to participation for parents should end before the infant gets its first immunization, which is according to the immunization schedule as soon as the infant is 6 weeks old. Registration must take place before the child has passed the age of 8 weeks, as this is the recommended time frame of the six-fold vaccination, which is sometimes given together with the rotavirus vaccine, which is recommended from 6 weeks on.

After registration, parents receive emails with further information depending on the age of the infant. Parents of unborn babies indicate the expected date of birth. They receive an email asking them to state the actual date of birth 3 weeks after this date. Parents of babies already born get invited to answer the first questionnaire as soon as their baby is 6 weeks old. The electronic case report forms will be stored in the Research Electronic Data Capture (REDCap) system (19, 20). Before the first question, parents get informed about the use and security of their data and their option to withdraw their consent to the use of their data. The first questionnaire in which parents get asked for their personal data, such as names and addresses, is kept in a database that is separate from the child’s health questions. A link between both databases is only possible via record linkage, with the email address being essential for sending invitation emails.

Parents will be interviewed via online case report forms at two time points. The first is before the first recommended vaccination, as mentioned before. The second time point occurs when the infant is 12 weeks old, as this is the time when the recommended time slot for the first immunization is over (Table 1). By this time, parents will have made decisions regarding whether and how to immunize their infant. If parents do not answer a questionnaire, they receive up to five reminder emails, which are sent every 5 days.

**Table 1:** Invitation and visit schedule of INFORMed study.

Separate visits	(V-1)	(V0)	V1	V2
Age of observed child (months)	Unborn*	<1 ½*	1 ½*	3
Informed consent	X	X	X	
True date of birth		X		
Attitude toward vaccination (C7C), demographic data, health status of the parents and siblings, and their vaccination			X	
Type of information, source of vaccination information, influencing factors, and trustworthiness of information sources			X	X
Details about vaccination advice and vaccination status				X

*Registration possible.

### Quality control

The electronic capture allows logical consistency tests and in case of incomplete or unclear responses recognized during statistical monitoring, direct clarification with parents is undertaken.

### Patient and public involvement

The questions of the case report forms are based on a qualitative study with young parents. However, the wishes of young parents are not only considered in protocol development, but are a fundamental focus of this investigation, because the information needs are evaluated not from physicians, but from parents’ perspective.

In addition, participating parents are invited to regular meetings during the study to report on their experience with the study and to offer suggestions.

### Measurements

The main question of this study is if vaccine-hesitant parents with negative attitudes wish less vaccine advice, as reported. To elaborate this further, we evaluate if and what kind of vaccination advice has been given to the parents and what was missing in their opinion. To analyze the type of vaccination advice, the case report form at the first visit includes questions about:

- The attitude toward vaccination will be assessed at the first visit using the C7C Scale (21). At the same time, confounding factors such as demographic data, health status of the parents and siblings, their vaccination status, and experience with vaccination will be assessed.
- Type of information based on a qualitative study (2) (e.g., effect and side effects, incidence and severity of the disease being vaccinated against, and information on divergent strategies)
- Source of vaccine information (Internet, books, circle of friends, midwife, and non-medical practitioner)
- Previous concrete understanding of the vaccination advice and strategy
- Influence of professionals and of the social environment
- Trust in professionals, social environment, and possible counseling centers

The second case report form at the age of 12 weeks (visit 2) contains the same questions, except the attitude toward vaccination but supplemented by information on:

- The professional who gave the advice (e.g., pediatrician, general doctor, and additional or other specialties)
- Details about vaccination advice (separate appointment/in the context of prevention examination/only at the vaccination procedure/information by a counseling interview/in writing; which brochures were delivered, duration of vaccination advice, fulfillment of the parental expectations, change of vaccination decision by the advice given and in what way, and influence of vaccination decision on further medical care)
- Other sources of information (e.g., Internet, books, circle of friends, midwife, and non-medical practitioner)
- The actual action: vaccination status at 12 weeks.

### Primary study endpoint

The primary study endpoint is the wish for vaccine advice before the first recommended period of vaccination, which is related to the initial attitude/hesitancy toward vaccinations.

### Secondary study endpoints

**Figure 1:**
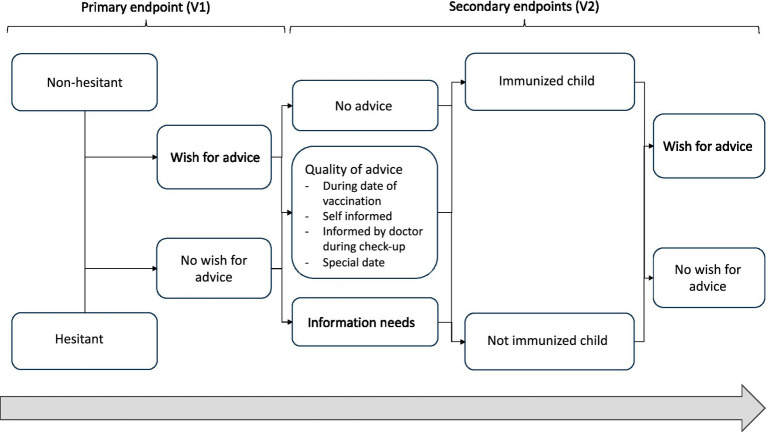
Schedule of shared decision-making and hence possible analyses of groups of new parents according to their attitude toward vaccination and actual action.

Secondary endpoints are the description of the scope of vaccination advice (such as advice received on the vaccination date, self-informed, informed by a doctor during a checkup, or at a special appointment); the perceived direction of the advice (e.g., non-hesitant or hesitant or undefined regarding vaccination); and the identification of influencing factors and suggestions for improvement. In addition, the need for information among four groups of parents will be described, along with the information content (Figure 1):

(a) Non-hesitant to vaccination and immunized child at 12 weeks of age
(b) Non-hesitant to vaccination but not immunized child at 12 weeks of age
(c) Hesitant to vaccination and not immunized child at 12 weeks of age
(d) Hesitant to vaccination but immunized child at 12 weeks of age.

### Sample size calculation

The sample size calculation is based on the data from the Federal Center for Health Education (BZgA) (22) in Germany. According to a cross-sectional survey conducted by the BZgA involving parents with children of various ages (n = 1.153), parents who (tend to) reject vaccinations want less often more information (4%) than parents who (tend to) support vaccinations (25%), which corresponds to an odds ratio of 8. The survey reports that 7% of parents are hesitant and tend to reject vaccination.

Based on these assumptions, and calculated using a Fisher’s exact test with a power of 80% and a two-tailed significance level of 5%, a minimum of 378 parents is needed, with 28 (7%) of them identified as hesitant. Considering the possibility that some surveys could be incomplete, at least 400 parents are needed.

### Study duration

The recruiting period for key persons and families is set for 2 years until the end of 2025 but may be extended depending on the success of the recruitment strategies of this group, which can be difficult to reach because of their vulnerable life stage.

### Analysis plan

After sample size completion, two groups (hesitant versus non-hesitant) will be made based on their initial attitude toward vaccination assessed using the C7C questionnaire. Using a chi-square test, the primary research question, as to whether hesitant people have a lesser proportion of wish for vaccine advice, can be answered in a confirmatory and prospective way for new parents. All other secondary analyses are explorative. With regression analyses, we will try to explore the various attitudes and reasons for both vaccination and vaccine advice. Further secondary endpoints such as results of information content, trustworthiness of information of different professions, the perceived influence, and suggestions for improvement will be reported as descriptive statistics and divided by the four groups (hesitant and not vaccinated, hesitant and vaccinated, non-hesitant and not vaccinated, and non-hesitant and vaccinated) as described above. All details are defined in a statistical analysis plan prior to the analyses.

### Dealing with bias and limitations

In contrast to existing surveys regarding vaccination attitudes, this study prospectively investigates attitude, change of attitude, and actual implementation of vaccination in the most important age group for this topic, namely, parents of newborns. As the first vaccination is recommended within a few weeks after birth, the examination of new parents poses some difficulties. For example, parents may not be willing to take part in a study during the probably stressful period following a birth. Furthermore, one group of parents (e.g., vaccine-hesitant or non-vaccine-hesitant) could participate more. Therefore, we assess the vaccination attitude of the parents in the beginning to adjust the group size if necessary.

Based on the chosen study period, attrition bias is possible if families do not answer the second questionnaire after vaccination. Nevertheless, the intentions and wishes they offered in the first questionnaire will be mentioned in the analysis of the primary outcome. Parents are only included if they have answered the first survey completely. Drop-out rates can be evaluated exploratorily according to the parents’ intentions.

To reduce detection bias, the groups and the statisticians will be blinded toward the outcomes as described before.

One limitation of the study is the exclusive view of the parents without considering the influence of the physicians. This may be a strength, too, because parents could be more open, but further studies that address physicians’ viewpoints will be necessary. This study does, however, also assess the attitudes of the participating physicians using a modified version of the 7C questionaire (23). Depending on the findings of this exclusively observational design, further studies that include interventions may follow. The exclusively observational design reduces self-reporting bias because only the parents’ viewpoints are considered. Vaccination uptake will be reported by the parents, and this carries a risk of bias. This risk will be reduced by the detailed query of vaccinations, which asks for the date, vaccine name, and diseases against which vaccinations are given.

Furthermore, the parents may feel under pressure to answer the questionnaires and may answer incorrectly. This risk of bias primarily affects the secondary endpoints and can be addressed by including the timing of responses in the analysis. As in this first stage of life, many vaccination dates are recommended, and rapid response from the parents is necessary. However, parents may forget to answer because of lack of sleep or other difficulties associated with the first months of an infant’s life, so reminding can be helpful to keep the participation rate high.

As some parents may visit different offices and physicians with different attitudes toward vaccination, the study may have to deal with performance bias. To enhance external validity in this context, physicians are requested to register their offices, while parents are asked to document their treating physicians.

In addition to the parental attitudes, this study also investigates the vaccine advice received, from the parental perspective, and the actual vaccinations given. Therefore, unconsidered covariates could be investigated.

## Discussion

This study addresses a very important population by clarifying whether vaccine-hesitant parents are generally less interested in vaccine advice (1). Furthermore, it can provide useful insight into the information wishes and needs of new parents. It is characterized not only by the fact that it deals with the population group most likely to come into contact with vaccinations, but also by the fact that it takes into account the three pillars of evidence-based medicine - science, patient and doctor - and their interactions, in this case from the parents’ perspective. These results can enhance information strategies, especially for new parents, and may give information on the different needs of vaccine-hesitant and non-hesitant parents.

However, this study has to deal with some difficulties, too. As research into vaccines continues to progress, the recommendations for childhood vaccinations are also constantly changing. This leads to parents being confronted with changing information, which may influence the comparability of the data. Particularly, the current recommendation for respiratory syncytial virus (RSV) vaccination in German newborns could influence the results, as the prospective study design cannot be realized for this vaccine if 3-day-old newborns receive vaccinations in the hospital before their first visit to a pediatrician (24). As the recommendation for those vaccinations depends on the month of birth, it may be necessary to include more families to be able to compare their strategies. On the other hand, there are legal and economic uncertainties with regard to the practical implementation of this recommendation, meaning that precise planning is not possible at the present time.

Another difficulty is finding participants for the study. Recruitment started in August 2023. Up to now, 42 parents have completed the second visit, the recruitment per month is far lower than expected, and the number of participating vaccine-hesitant parents is much higher than the non-hesitant parents. Therefore, the number of participating offices must be expanded, and the recruitment of non-hesitant parents needs to be optimized. By now, promotion strategies seem mostly to address vaccine-hesitant parents. Consequently, the wording and appearance of the flyer and other promotion materials will be designed with feedback from hesitant and non-hesitant parents. Other strategies could include multiplicators that give parents the possibility to register during their contact with the multiplicator or focusing on obstetric clinics as multiplicators. However, the current recruitment delay is due to initial organizational difficulties. We are currently working on eliminating these difficulties and developing advertisement strategies. We welcome further supporting offices, which is one of the reasons for submitting this protocol for publication.

Previous research has shown that balanced leaflets help to improve decision-making in parents and do not influence their decision (25). A more detailed understanding of the shared decision process, considering both the *a priori* and *a posteriori* wish for advice, the experienced advice, and vaccine administration as a common intervention, may help reduce vaccine hesitancy and improve public healthcare outcomes. Based on the findings of this study, an intervention that addresses parents’ needs for advice can be designed and tested. Furthermore, the results may help to secure resources in pediatric settings and improve vaccine acceptance if parents get the information they need. The findings about attitudes, interventions, and subsequent actions can be extrapolated to other medical settings such as medication, inpatient stays, or surgeries.
